# Heparin suppresses FoxO1/pFoxO1 signaling axis in vascular smooth muscle cells

**DOI:** 10.1016/j.bbrep.2025.101954

**Published:** 2025-02-19

**Authors:** Nafiseh Shokri, Mohammad Elahimanesh, Masoomeh Bakhshandeh, Mohammad Najafi

**Affiliations:** a Clinical Biochemistry Department, Faculty of Medical Sciences, Iran University of Medical Sciences, Tehran, Iran; b Molecular and Cellular Research Center, Iran University of Medical Sciences, Tehran, Iran; c Clinical Biochemistry Department, Faculty of Medical Sciences, Shahid Beheshti University of Medical Sciences, Tehran, Iran

**Keywords:** FoxO1, pFoxO1, VSMCs, Heparin

## Abstract

**Background:**

The atherosclerosis process relates to the dysfunction of different cells in the plaque microenvironment. The vascular smooth muscle cells (VSMCs) play an important role in atherosclerosis. Since the FoxO family is reported to control cell growth, thus the aim of this study was to investigate the effects of Heparin, Betulinic acid, and Ibrutinib on the FoxO1/pFoxO1 axis in vascular smooth muscle cells.

**Methods:**

The VSMCs were cultured in DMEM-F12 medium and, were treated with Heparin (30IU), Betulinic acid (60 μM), and Ibrutinib (2 μM) in the 24 and 48-h periods. The FoxO1 gene expression level was identified using the RT-qPCR technique. The FoxO1 and pFoxO1 protein values were measured by the Western blot technique.

**Results:**

The FoxO1 gene expression levels were reduced significantly in the cellular groups treated with Heparin, Betulinic acid, and Ibrutinib (P < 0.05) in both periods. Moreover, the pFoxO1 and FoxO1 protein values were decreased by Heparin, and Betulinic acid in the VSMCs.

**Conclusion:**

Heparin suppressed the FoxO1/pFoxO1 signaling axis in vascular smooth muscle cells (VSMCs). Furthermore, it modulated the effects of Betulinic acid and Ibrutinib.

## Highlights

• Heparin suppressed the pFoxO1/FoxO1 axis in VSMCs.
•Betulinc acid suppressed the pFoxO1/FoxO1 axis in VSMCs.
•Heparin affected the roles of Betulinc acid and Ibrutinib on VSMCs.

## 1 Introduction

Cellular dysfunctions and extracellular matrix changes progress the atheromatous plaques in the sub-endothelial space of vessels [1,2]. The vascular smooth muscle cells (VSMCs) are key players that proliferate and migrate abnormally in the plaque microenvironment contributing to plaque progression [3,4]. Some studies reported that the O1 subfamily of Forkhead box protein (FoxO1), as a transcription factor (TF), has a crucial role in cellular signaling pathways. It is reported to relate to signaling pathways involved in apoptosis, proliferation, autophagy, PDGF-BB, TNF-α, and IGF1. It also inhibits the proliferation of vascular smooth muscle cells (VSMCs) [[5], [6], [7], [8]]. FoxO1 is expressed in many tissues and is regulated in response to different pathologic conditions [5,9]. Its function has been linked to some metabolic diseases, atherosclerosis, cancer, and cardiac hypertrophy [5,9,10]. Some studies reported that Heparin affects the accumulation of FoxO1 in the nucleus [11]. Heparin-binding growth factors showed to improve the growth of VSMCs [12]. On the other hand, some chemicals such as Betulinic acid and Ibrutinib affected cellular growth. Betulinic acid decreased the human dermal fibroblast senescent cells and induced the SIRT1/FoxO1 pathway in MCAO rats [13,14]. Ibrutinib is also reported to inhibit the growth of HER2^+^ breast cancer cell lines [15]. These reports indicated that Betulinic acid elevates cell growth while Ibrutinib suppresses cell expansion. The above studies also suggested that the effects of Heparin on the growth of VSMCs might follow via the FoxO1/pFoxO1 axis. Thus, the study of effects of Heparin could be further interpreted when it is simultaneously evaluated with other chemical compounds, such as Betulinic acid and Ibrutinib, that affect cell growth. It was also well known that PI3K-Act, mTOR, and AMPK signaling pathways through FoxO and NF-kappa B pathways affect cell growth [16,17]. Since in the previous report, Heparin affected the NF-kB signaling pathway in the VSMCs [18] thus FoxO1 axis was investigated in this study. We investigated the FoxO1 gene expression levels and the FoxO1/pFoxO1 protein values in the vascular smooth muscle cells treated with Heparin, Betulinic acid and Ibrutinib.

## 2 Materials and methods

### 2.1 Vascular smooth muscle cells (VSMCs)

The VSMCs (C591) were provided from the Tehran Pasteur Institute. The cells were cultivated in DMEM (Dulbecco's modified eagle media)-F12 (Cat. Number. BI-1011, Bioidea, Iran) containing 10 % Fetal Bovine Serum (FBS, Cat. Number. BI-1201, Bioidea, Iran) and 1 % penicillin–streptomycin (Pen/Strep, Cat. Number. BI-1203, Bioidea, Iran). The cell groups were included: Control (C), Heparin (H, 30IU, Cat. Number. H3149, Sigma, USA), Betulinic acid (B, 60 μM, (EC Number. 207-488-6)), Ibrutinib (I, 2 μM, (CAS Number: 936563-96-1)), Heparin (30IU) + Betulinic acid (60 μM) (H + B), and Heparin (30IU) + Ibrutinib (2 μM) (H + I). The values of Heparin (30IU), Betulinic acid (60 μM), and Ibrutinib (2 μM) were used on the cellular viability and cytotoxicity reports [18].

### 2.2 RNA extraction

Total RNA was prepared from VSMCs using the RNA extraction kit (Cat. Number: EX60311, Sinacolon, Iran) according to the producer's instructions in the 24- and 48-h periods. A nanodrop device was used to evaluate the RNA quantity.

### 2.3 cDNA synthesis

According to the producer's instructions (Yektatajhiz kit, Iran), RNA sample, buffer, RNAase, M-MLV, Oligonucleotide, Random hexamer, and dNTPs were used for the cDNA synthesis.

### 2.4 Real-time qPCR technique

SYBR Green PCR Master Mix (Cat. Number: YT2551, Yektatajhiz, Iran) and the cDNA sample were used in the RT-qPCR technique. The GAPDH gene was used as the reference gene. The gene primers are listed in Table 1.

**Table 1:** The primers.

Gene	Forward sequence	Reverse sequence
FoxO1	5′- TACGAGTGGATGGTCAAGAGCGTG -3′	5′-CTTGCCACCCTCTGGATTGAGCA- 3′
GAPDH	5′-CATGAGAAGTATGACAACAGCCT-3′	5′-AGTCCTTCCACGATACCAAAGT-3′

### 2.5 Western blot technique

The total protein was extracted from VSMCs using RIPA buffer (Cat. Number. DB9719, DNA Biotech, Iran) containing Phenyl methyl sulfonyl fluoride (PMSF) and enzymatic inhibitors. The supernatant was separated by centrifugation (13000 g) for 20 min (4 °C). Then, 3 μl was electrophoresed (90V) on SDS-PAGE (12 %) for 45 min. The protein spots were transferred into the PVDF membrane (Cat. Number. IPVH00010, Merck Millipore, Germany) for 60 min. The PVDF membrane was blocked with 4 % skim milk (Cell Signaling Technology, USA) for 60 min. According to the previous study [18], first, the blots of NF-κB and Beta-Actin (1:1000 v/v, Cell Signaling Technology, Beverly, MA, USA, Cat. Number. 4967s) were visualized on the PVDF membrane then, it was washed using Tris-buffered saline and were preserved at −80 °C. The Beta-Actin was applied as a reference protein to normalize all the target protein blots, which were obtained by re-using the PVDF membrane. To estimate FoxO1/pFoxO1 protein blots, the re-washed PVDF membrane incubated with primary pFoxO1 antibody (1:1000 v/v, Cat. Number. Ser256, Cell Signaling, USA), and primary FoxO1 antibody (1:1000 v/v, Cat. Number. C29H4, Cell Signaling, USA). Following overnight (4 °C), the PVDF membrane was incubated with a secondary antibody (1:2000 v/v, Cat. Number. 7074s, Cell Signaling Technology, Beverly, MA, USA) for 60 min at room temperature. Then, the membrane was washed with Tris-buffered saline containing 0.1 % Tween 20 (TBST), and was exposed to the Enhanced chemiluminescence (ECL).

### 2.6 Statistical analysis

The data were analyzed using SPSS (Version 27.0.1). Initially, the Shapiro-Wilk test was employed to assess the data distribution. The gene and protein changes between the cell groups were compared using the ANOVA test, and equal variances were assumed using the Tukey test. Protein linkages on heatmaps were determined with Euclidean method. The cycle threshold (CT) values for FoxO1 and housekeeping genes were identified in all the cellular groups treated during the 24- and 48-h periods. The 2^−ΔΔCT^ formula was applied to calculate the gene expression changes. The Image J software was utilized for the Western blot image analysis.

## 3 Results

### 3.1 FoxO1 gene expression levels reduced in the VSMCs treated with heparin, betulinic acid, and ibrutinib

**Fig. 1:**
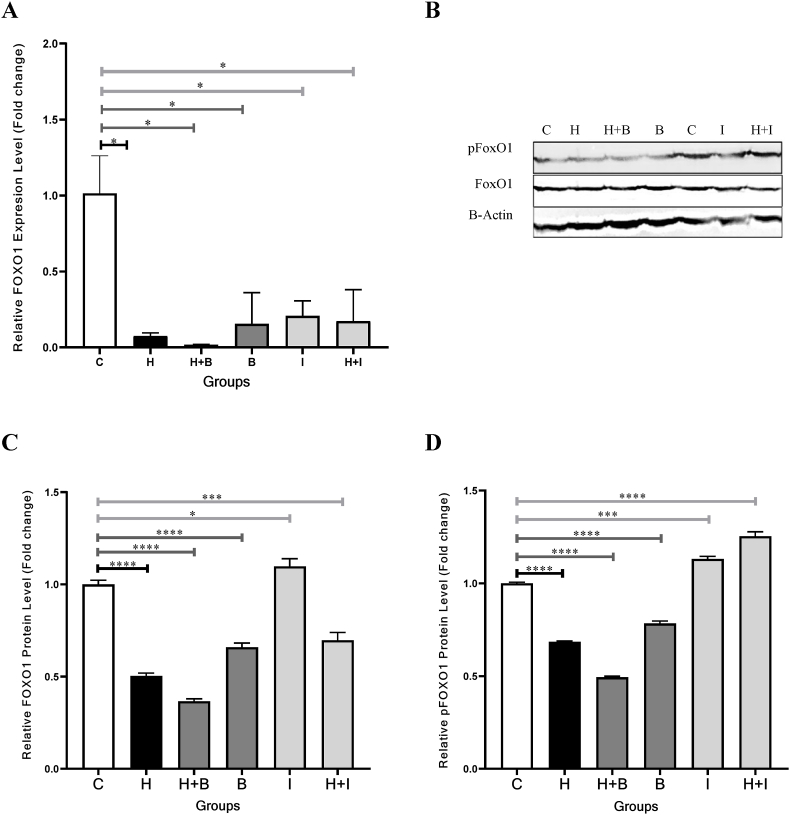
FoxO1 gene expression levels and FoxO1/pFoxO1protein values in VSMCs treated with Heparin, Betulinic acid, and Ibrutinib in 24-h period. **A,** All groups decreased significantly the FoxO1 gene expression levels. **B**, Western blot Images. The control (C) sample was repeated to evaluate the run repeatability (1 and 5 lanes). **C,** The FoxO1 protein values reduced significantly in VSMCs treated with Heparin, and Betulinic acid. **D,** The pFoxO1 protein values reduced in VSMCs treated with Heparin, and Betulinic acid. Control, C; Heparin, H; Betulinic acid, B; Heparin + Betulinic acid, H + B; Ibrutinib, I; Heparin + Ibrutinib, H + I. Data are repeated (n = 3) and presented as mean ± SD. ∗p < 0.05, ∗∗∗p < 0.001, ∗∗∗∗p < 0.0001.

**Fig. 2:**
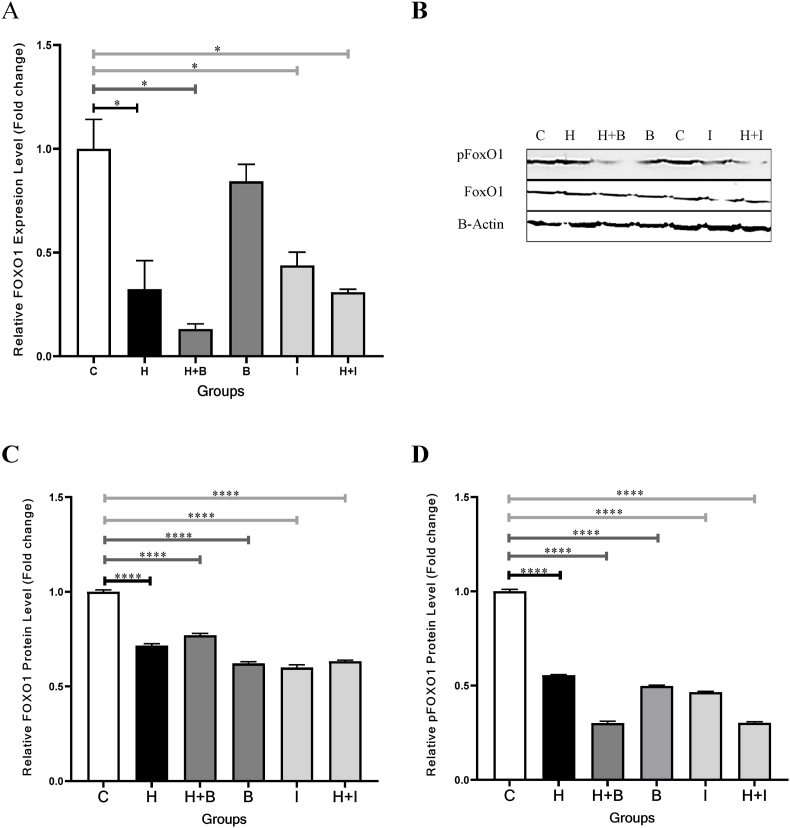
FoxO1 gene expression levels and FoxO1/pFoxO1protein values in VSMCs treated with Heparin, Betulinic acid, and Ibrutinib in 48-h period. **A,** Heparin and Ibrutinib decreased significantly the FoxO1 gene expression level. **B**, Western blot Images. The control (C) sample was repeated to evaluate the run repeatability (1 and 5 lanes). **C,** The FoxO1 protein values reduced significantly in all the groups. **D**, The pFoxO1 protein values reduced significantly in all the groups. Control, C; Heparin, H; Betulinic acid, B; Heparin + Betulinic acid, H + B; Ibrutinib, I; Heparin + Ibrutinib, H + I. Data are repeated (n = 3) and presented as mean ± SD. ∗∗p < 0.05, ∗∗∗p < 0.001, ∗∗∗∗p < 0.0001.

The FoxO1 gene expression levels reduced significantly in the groups treated with Heparin (H, P = 0.0329), Heparin + Betulinic acid (H + B, P = 0.0292), Betulinic acid (B, P = 0.0216), Ibrutinib (I, P = 0.0499), and Heparin + Ibrutinib (H + I, P = 0.0205) after 24 h as compared to the control (C) group (Fig. 1, A and Table 2). Moreover, similar results were observed after 48 h in the groups treated with Heparin (H, P = 0.0400), Heparin + Betulinic acid (H + B, P = 0.0134), Ibrutinib (I, P = 0.0361), and Heparin + Ibrutinib (H + I, P = 0.02) (Fig. 2, A and Table 2). The Heparin modulated the FoxO1 gene expression levels in cellular groups containing Betulinic acid (B, 24 and 48-h periods) and Ibrutinib (I, 48-h period) (P < 0.05). However, the FoxO1 gene expression level was not changed in the group treated with Heparin + Ibrutinib (H + I) as compared to Ibrutinib (I) in the 24-h period (P = 0.7) (Fig. 1, A).

**Table 2:** Gene and protein changes among the study cell groups.

	24-h Period						48-h Period					
	FoxO1 Expression Level, Fold Change (%)	P-Value	FoxO1 value, Fold Change (%)	P-Value	pFoxO1 value, Fold Change (%)	P-Value	FoxO1 Expression Level, Fold Change (%)	P-Value	FoxO1 value, Fold Change (%)	P-Value	pFoxO1 value, Fold Change (%)	P-Value
Control	1(100)		1(100)		1(100)		1(100)		1(100)		1(100)	
Heparin	0.1 (10)	**<0.05**	0.5 (50)	**<0.0001**	0.72 (72)	**<0.0001**	0.32 (32)	**<0.05**	0.72 (72)	**<0.0001**	0.58 (58)	**<0.0001**
Heparin + Butulinic Acid	0.02 (2)	**<0.05**	0.38 (38)	**<0.0001**	0.49 (49)	**<0.0001**	0.12 (12)	**<0.05**	0.75 (75)	**<0.0001**	0.28 (28)	**<0.0001**
Butulinic Acid	0.18 (18)	**<0.05**	0.63 (63)	**<0.0001**	0.76 (76)	**<0.0001**	0.89 (89)	>0.05	0.6 (60)	**<0.0001**	0.49 (49)	**<0.0001**
Ibrutinib	0.22 (22)	**<0.05**	1.07 (107)	**<0.05**	1.1 (110)	**<0.001**	0.46 (46)	**<0.05**	0.58 (58)	**<0.0001**	0.48 (48)	**<0.0001**
Heparin + Ibrutinib	0.2 (20)	**<0.05**	0.7 (70)	**<0.001**	1.25 (125)	**<0.0001**	0.3 (30)	**<0.05**	0.61 (61)	**<0.0001**	0.29 (29)	**<0.0001**

### 3.2 FoxO1 protein expression levels decreased in the VSMCs treated with heparin, and betulinic acid

The FoxO1 protein expression levels reduced significantly in the Heparin (H, P < 0.0001), Heparin + Betulinic acid (H + B, P < 0.0001), Betulinic acid (B, P < 0.0001), Heparin + Ibrutinib (H + I, P < 0.001) treated groups after 24 h as compared to the control (C) group (Fig. 1, B (Supplementary file 1) and C, Table 2). Ibrutinib also increased significantly the FoxO1 protein expression level in the VSMCs during 24 h (P < 0.05) (Fig. 1, C). The FoxO1 protein expression levels reduced significantly after 48 h in Heparin (H, P < 0.0001), Heparin + Betulinic acid (H + B, P < 0.0001), Betulinic acid (B, P < 0.0001), Ibrutinib (I, P < 0.0001), Heparin + Ibrutinib (H + I, P < 0.0001) treated groups as compared to the control (C) group (Fig. 2, B (Supplementary file 1) and C, Table 2). By adding Heparin to the culture medium containing Betulinic acid and Ibrutinib, the FoxO1 protein expression levels were further reduced in the 24-h period.

### 3.3 pFoxO1 protein decreased in the VSMCs treated with heparin, and betulinic acid

The pFoxO1 protein decreased significantly in the groups treated with Heparin (H, P < 0.0001), Heparin + Betulinic acid (H + B, P < 0.0001), and Betulinic acid (B, P < 0.0001) after 24 h as compared to the control (C) group (Fig. 1, B (Supplementary file 1) and D, Table 2). However, a significant increase was observed in the groups treated with the Ibrutinib (I, P < 0.001) and Heparin + Ibrutinib (H + I, P < 0.0001). The pFoxO1 protein values also decreased significantly in the Heparin (H, P < 0.0001), Heparin + Betulinic acid (H + B, P < 0.0001), Betulinic acid (B, P < 0.0001), and Heparin + Ibrutinib (H + I, P < 0.0001), Ibrutinib (I, P < 0.0001) treated groups after 48 h as compared to the control (C) group (Fig. 2, B (Supplementary file 1) and D, Table 2). By incorporating Heparin into the culture medium containing Betulinic acid, the pFoxO1 protein values were further reduced in both the 24 and 48-h periods (P < 0.05).

### 3.4 pFoxO1/FoxO1 changes in the VSMCs treated with heparin, ibrutinib, and betulinic acid

**Fig. 3:**
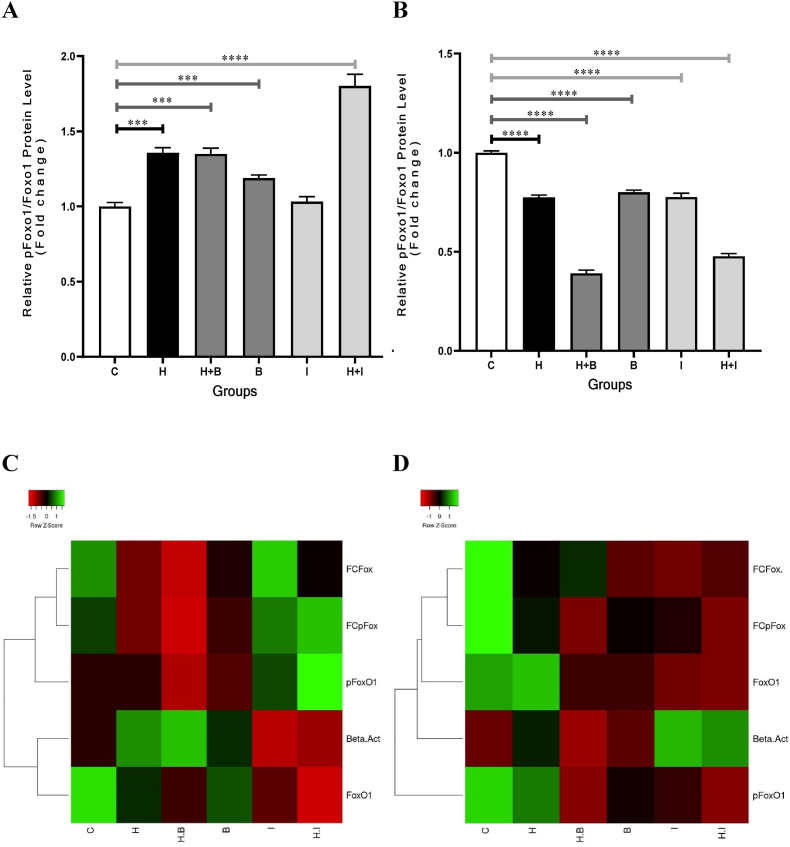
pFoxO1/FoxO1 changes in VSMCs treated with Heparin, Betulinic acid, and Ibrutinib. **A**, 24-h period: Heparin increased significantly the pFoxO1/FoxO1 ratios in the VSMCs treated with Betulinic acid and Ibrutinib. **B**, 48-h period: The pFoxO1/FoxO1 ratios reduced significantly in all the cellular groups. Protein linkages were observed between FoxO1 Fold changes (FCFox) and pFoxO1 Fold changes (FCpFox) in the 24-h (**C**) and 48-h (**D**) periods. Control, C; Heparin, H; Betulinic acid, B; Heparin + Betulinic acid, H + B; Ibrutinib, I; Heparin + Ibrutinib, H + I. Data are repeated (n = 3) and presented as mean ± SD. ∗∗∗p < 0.001, ∗∗∗∗p < 0.0001.

The pFoxO1/FoxO1 ratios increased in all the groups as compared to control in the 24-h period (Fig. 3, A). It also reduced in all the groups treated in the 48-h period (Fig. 3, B). Furthermore, the protein linkages between pFoxO1 and FoxO1 fold changes showed that the changes of pFoxO1 protein values related to FoxO1 protein values in both 24 and 48-h periods (Fig. 3C and D).

## 4 Discussion

The progression of atherosclerosis relates to the dysfunction of different cells and extracellular matrix remodeling in the plaque microenvironment. The vascular smooth muscle cells (VSMCs) play an important role in atherosclerosis and vessel restenosis [19,20]. FoxO family is reported to control cellular growth through the proliferation, migration, and apoptosis processes in the VSMCs [5]. In the previous study [18], the results showed that Heparin (30IU) activates the NF-κB signaling pathway. The purpose of this study was to investigate the pFoxO1/FoxO1 axis in the vascular smooth muscle cells treated with Heparin. Furthermore, Betulinic acid and Ibrutinib were used for their roles in cell growth, so the role of Heparin might be further determined. Some studies reported that the FoxO family acts primarily as a transcription factor but it can have “off-target” effects as a co-regulator or interactor with other proteins [21]. FoxO1 as the number of this family plays a vital role and is highly expressed in the vascular and endothelial cells [19,20]. Moreover, the FoxO family is involved in oxidative stress, metabolism, cell cycle, growth, and death in the context of cardiac function [10,19,22]. Several studies reported that the FoxO family is elevated in atherosclerosis and diabetic cardiomyopathy [20,[23], [24], [25]]. Since the phosphorylation of FoxO1 controls its role in cell growth, thus the changes of FoxO1/pFoxO1 axis relate to molecular pathogenesis in some diseases.

Studies showed that the roles of Heparin conjugates and derivatives on angiogenesis, cell adhesion, and migration relate to the cell characteristics, chemical structure, and duration of exposure periods. Heparin has also been shown to have anti-proliferative effects while some studies found that it effectively promotes cell growth [[26], [27], [28], [29], [30], [31], [32]]. In the hypothalamus neurons of wild-type mice, Heparin reduced the FoxO1 phosphorylation enhancing nuclear translocation [33]. Heparin also activates some receptors such as anaplastic lymphoma kinase (ALK) [34]. A study reported that mediating the TMEM184A receptor by Heparin modulates the ERK pathway in the VSMCs [35]. Furthermore, Heparin through the inhibition of FoxO1 promotes AgRP neurons regulating food intake [36]. Some studies also reported that Heparin causes the nuclear aggregation of FoxO1 in the decidualized hESCs [37]. Other studies, however, reported the opposite results [10,19]. Our results showed that Heparin suppresses the FoxO1 gene expression levels and FoxO1/pFoxO1 protein values. Moreover, the protein linkage analyses showed that the changes of pFoxO1 are related to the FoxO1 values so the study suggested the suppression of FoxO1 protein values affects directly pFoxO1 values as usual cellular feedback.

Betulinic acid (B) has different pharmacological roles in human cells. It alleviates cellular senescence in dermal fibroblasts by inhibiting the type 1 IFN signaling pathway [13]. Moreover, Betulinic acid induced the SIRT1/FoxO1 pathway to reduce autophagy and enhance brain damage [38]. Our results showed that Betulinic acid reduces significantly both the FoxO1 gene expression levels and FoxO1/pFoxO1 protein values [16]. Ibrutinib is a tyrosine kinase inhibitor and has been reported to diminish cellular migration and proliferation [18,39,40]. Some studies found that Ibrutinib can reduce platelet aggregation in arterial blood flow [41]. The combination of AZD8055 and Ibrutinib increased significantly FoxO1 activity in CLL cells [42]. Our results showed that Ibrutinib had a significant inhibitory effect on the FoxO1 gene expression levels. However, the FoxO1 protein value was high in the short time period.

In conclusion, our results showed that Heparin (30 IU) decreased the FoxO1 protein and gene expression levels. Moreover, the changes in pFoxO1 protein values are related to the FoxO1 protein values. On the other hand, adding Heparin into the culture medium including Betulinc acid and Ibrutinib modulated the pFoxO1 and FoxO1 protein and gene expression levels. However, the limitations of study to evaluate the effects of Heparin on the cellular growth, apoptosis and longevity must be investigated through cross-talking the FoxO1/FoxO1 axis with other signaling pathways. The cellular longevity might be investigated on mitochondrial function and oxidant balance. Furthermore, investigating downstream targets of FoxO1/pFoxO1 and validation should also be evaluated in vivo models of atherosclerosis and vascular diseases in the future.

## CRediT authorship contribution statement

**Nafiseh Shokri:** Writing – original draft, Investigation, Data curation. **Mohammad Elahimanesh:** Writing – original draft, Data curation. **Masoomeh Bakhshandeh:** Writing – original draft, Data curation. **Mohammad Najafi:** Writing – review & editing, Writing – original draft, Visualization, Supervision.

## Ethics approval and consent to participate

Not applicable.

## Consent for publication

Not applicable.

## Availability of data and materials

The data generated and analyzed during the current study are available from the corresponding author on reasonable request.

## Human and animal rights

No animals/humans were used for this study.

## Funding

It is supported by 10.13039/100012021Iran University of Medical Sciences (No. 25166).

## Declaration of competing interest

The authors declare the following financial interests/personal relationships which may be considered as potential competing interests: No.

## Data Availability

Data will be made available on request.
