# Supplementary material for: Heparin suppresses FoxO1/pFoxO1 signaling axis in vascular smooth muscle cells
Source: Biochem Biophys Rep. 2025 Feb 19;41:101954. doi: 10.1016/j.bbrep.2025.101954 (PMC11880713; doi:10.1016/j.bbrep.2025.101954)
Supplement: Multimedia component 1 [file mmc1.docx]

**A**


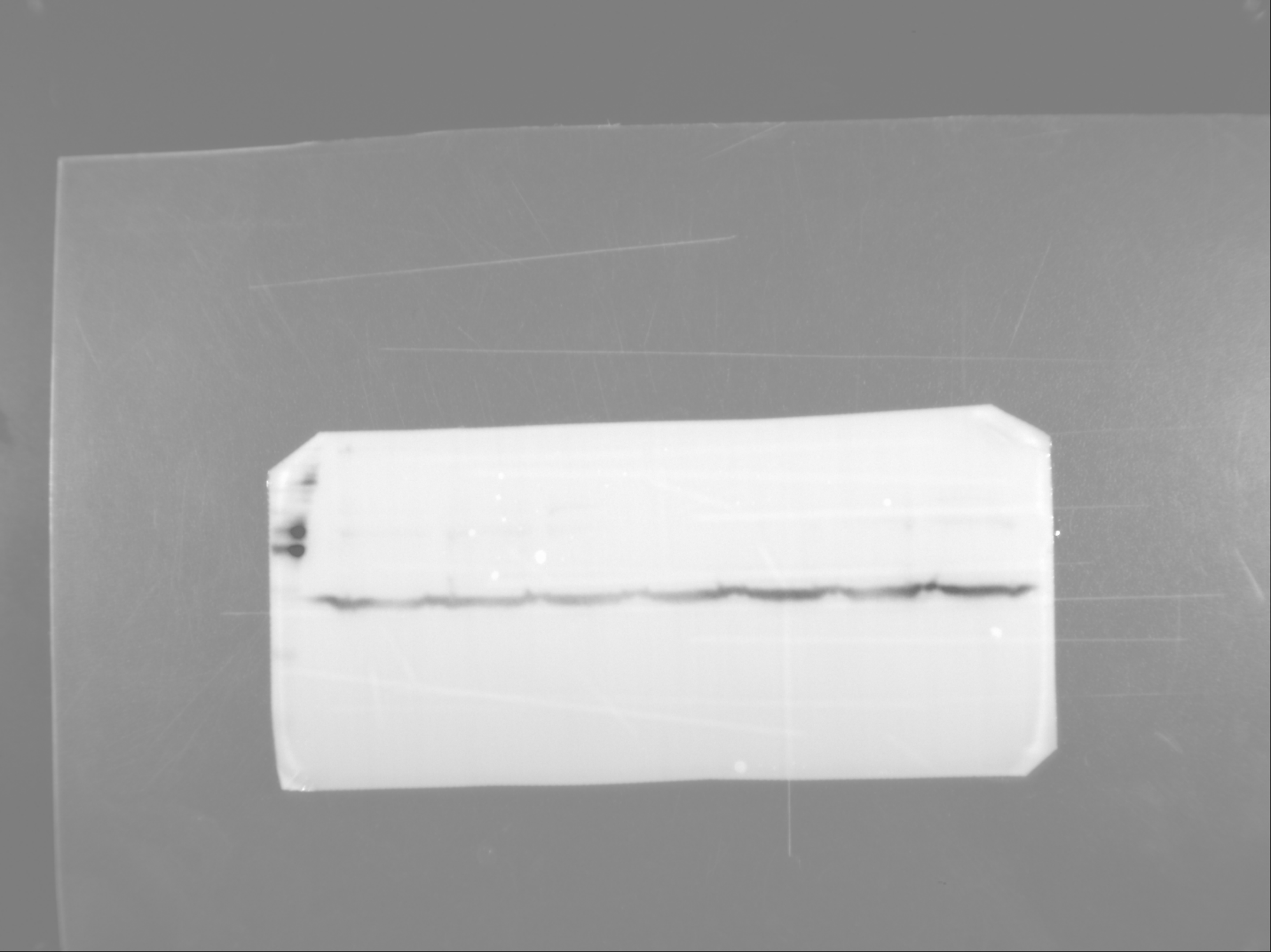


| 1 | 2 | 3 | 4 | 5 | 6 | 7 | 8 |
| --- | --- | --- | --- | --- | --- | --- | --- |

**A: pFoxO1 protein expression levels in vascular smooth muscle cells (VSMCs) in 24-hour period. From left to right: 1. Ladder, 2. Control, 3. Heparin 30IU, 4. Heparin 30IU + Betulinic acid 60µM, 5. Betulinic acid 60µM, 6. Control, 7. Ibrutinib 2µM, 8. Heparin 30IU + Ibrutinib 2µM .**

**B**


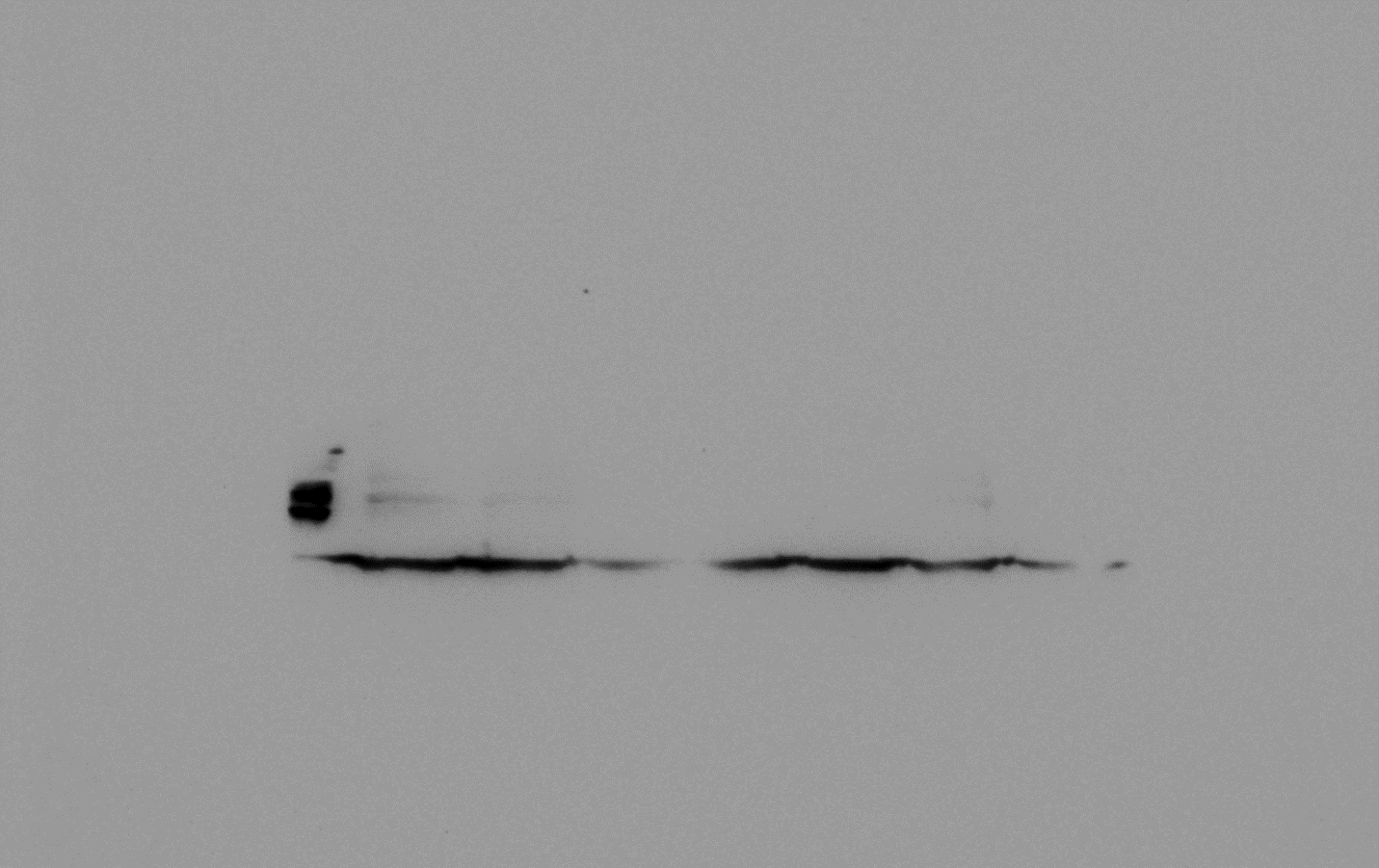


**B: pFoxO1 protein expression levels in vascular smooth muscle cells (VSMCs) in 48-hour period. From left to right: 1. Ladder, 2. Control, 3. Heparin 30IU, 4. Heparin 30IU + Betulinic acid 60µM, 5. Betulinic acid 60µM, 6. Control, 7. Ibrutinib 2µM, 8. Heparin 30IU + Ibrutinib 2µM.**

**C**


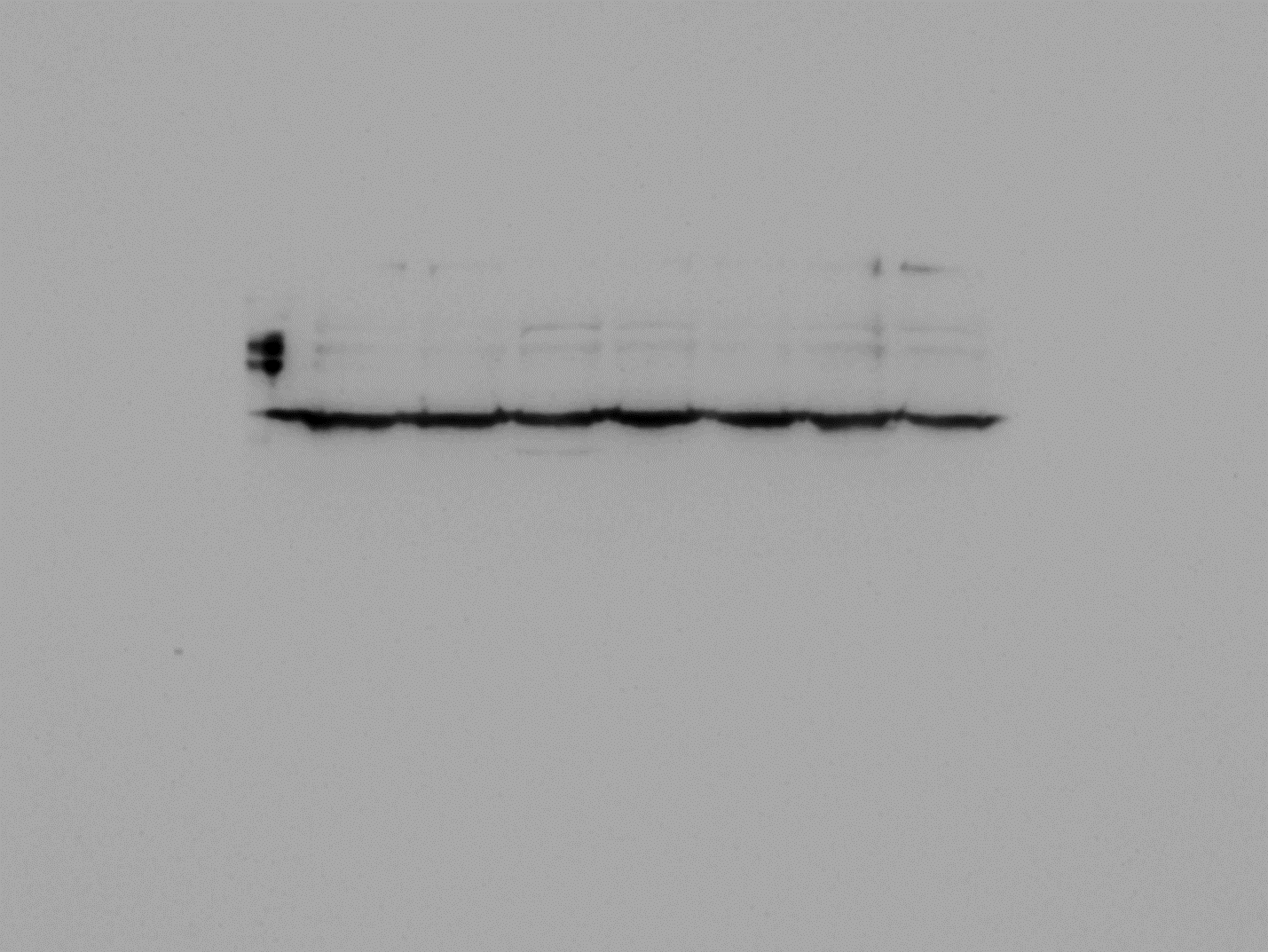


**C: FoxO1 protein expression levels in vascular smooth muscle cells (VSMCs) in 24-hour period .From left to right: 1. Ladder, 2. Control, 3. Heparin 30IU, 4. Heparin 30IU + Betulinic acid 60µM, 5. Betulinic acid 60µM, 6. Control, 7. Ibrutinib 2µM, 8. Heparin 30IU + Ibrutinib 2µM .**

**D**


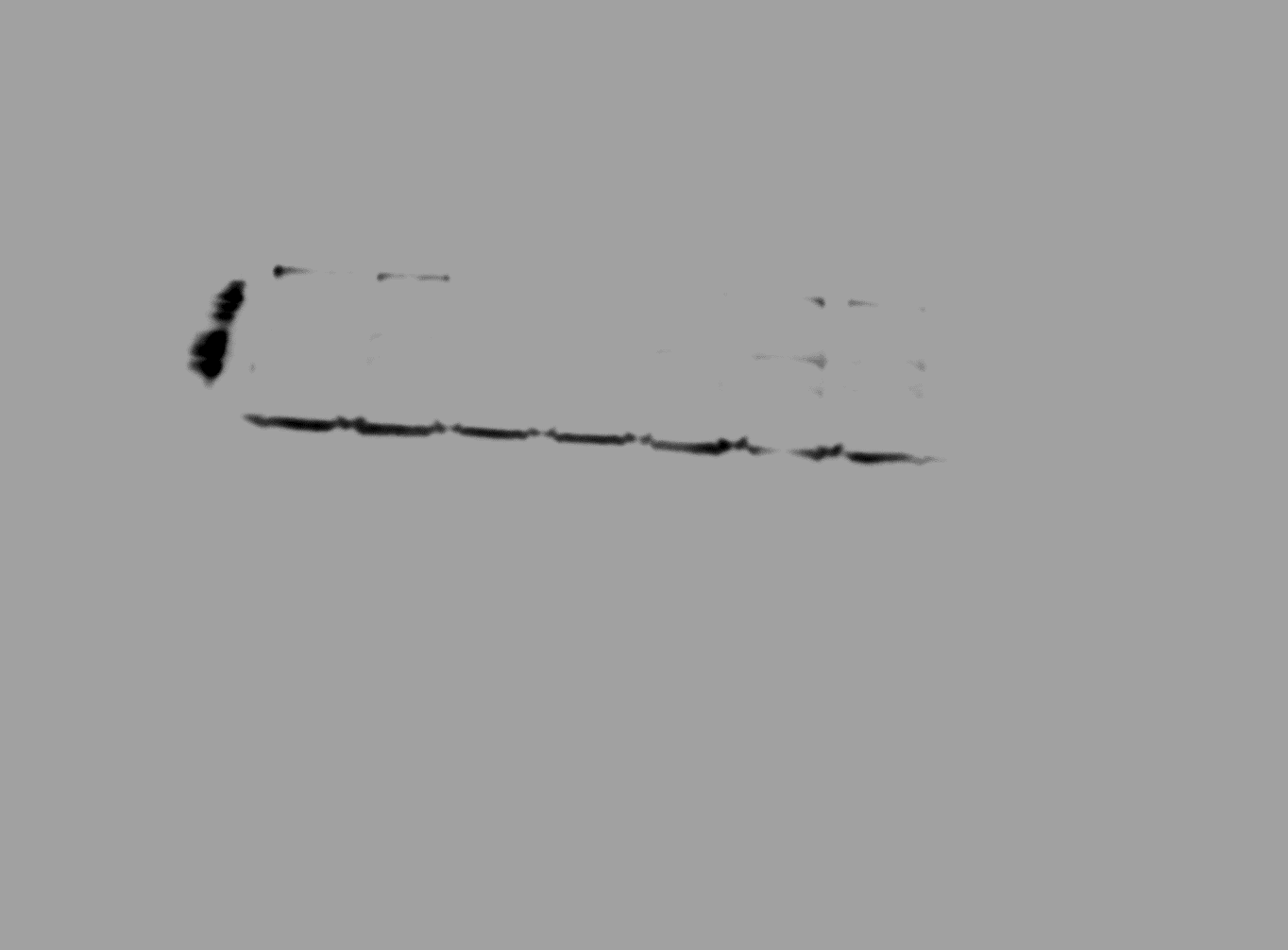


**D: FoxO1 protein expression levels in vascular smooth muscle cells (VSMCs) in 48-hour period. From left to right: 1. Ladder, 2. Control, 3. Heparin 30IU, 4. Heparin 30IU + Betulinic acid 60µM, 5. Betulinic acid 60µM, 6. Control, 7. Ibrutinib 2µM, 8. Heparin 30IU + Ibrutinib 2µM .**
